# A chromosome-level genome assembly of the pig-nosed turtle (*Carettochelys insculpta*)

**DOI:** 10.1038/s41597-024-03157-8

**Published:** 2024-03-23

**Authors:** Ye Li, Yuxuan Liu, Jiangmin Zheng, Baosheng Wu, Xinxin Cui, Wenjie Xu, Chenglong Zhu, Qiang Qiu, Kun Wang

**Affiliations:** 1 Shaanxi Key Laboratory of Qinling Ecological Intelligent Monitoring and Protection, School of Ecology and Environment, Northwestern Polytechnical University, Xi’an, 710072 China; 2 Guangdong Key Laboratory of Animal Conservation and Resource Utilization, Institute of Zoology, Guangdong Academy of Sciences, Guangzhou, 510260 China

**Keywords:** Genome, Sequencing

## Abstract

The pig-nosed turtle (*Carettochelys insculpta*) represents the only extant species within the Carettochelyidae family, is a unique Trionychia member fully adapted to aquatic life and currently facing endangerment. To enhance our understanding of this species and contribute to its conservation efforts, we employed high-fidelity (HiFi) and Hi-C sequencing technology to generate its genome assembly at the chromosome level. The assembly result spans 2.18 Gb, with a contig N50 of 126 Mb, encompassing 34 chromosomes that account for 99.6% of the genome. The assembly has a BUSCO score above 95% with different databases and strong collinearity with Yangtze giant softshell turtles (*Rafetus swinhoei*), indicating its completeness and continuity. A total of 19,175 genes and 46.86% repetitive sequences were annotated. The availability of this chromosome-scale genome represents a valuable resource for the pig-nosed turtle, providing insights into its aquatic adaptation and serving as a foundation for future turtle research.

## Background & Summary

The pig-nosed turtle is a remarkable and unique organism within the world of Chelonians, standing as the only extant species within the genus *Carettochelys*^1^. Known for its distinct pig-like nose, paddle-shaped fore flippers adorned with claws, and a shell that can reach up to 50 cm in length in females, this species has evolved a fascinating suite of phenotype that set it apart from other turtles^1^. The closest living relative of the softshell turtle, pig-nosed turtle thrives in habitats such as large rivers, swamps, lagoons, and freshwater environments found in southern Lrian Jaya (Indonesia), southern Papua New Guinea, and the major river systems of the northwestern Northern Territory in Australia^2,3^.

Unfortunately, despite its evolutionary and ecological significance, the pig-nosed turtle is facing a high risk of extinction due to a combination of habitat loss, overexploitation for food and the pet trade, and other anthropogenic pressures^4^. These threats have caused a precipitous decline in its wild population, leading to its classification as “Insufficiently Known” in the 1982 Red Data Book^5^ and as “Vulnerable” in the 1996 Red List^6^. These conservation challenges highlight the urgent need for comprehensive molecular data to inform effective protection strategies.

Despite extensive research has been conducted on turtle sex determination and other traits^7,8^, the research for pig-nosed turtle, as a unique Trionychia member fully adapted to aquatic life, has not yet been systematically analyzed due to the lack of a high-quality genome data. In this study, we present a chromosome-level draft genome of the pig-nosed turtle (~2.18 Gb) with a contig N50 of 126 Mb achieved through advanced PacBio high-fidelity (HiFi) sequencing and chromosome conformation capture (Hi-C) sequencing techniques. Conserved core genes (BUSCO score) and genome synteny confirmed the continuity and accuracy of the assembly, representing the highest quality Testudines genome assembled to date. Overall, the high-quality genome serves as a valuable resource for future research on chelonian evolution and conservation.

## Methods

### Sample collection and sequencing

An artificially bred pig-nosed turtle was obtained from an aquarium in Xiong County, Hebei Province, China. The turtle was anesthetized, and its abdominal cavity was exposed to collect tissue samples, including liver, muscle, kidney, spleen, trachea, and lungs. These samples were promptly frozen in liquid nitrogen and stored at −80 °C. For genomic DNA extraction, muscle tissues were used for both short-reads sequencing and HiFi sequencing, while liver was used for Hi-C sequencing. Additionally, various tissues including muscle, liver, kidney, spleen, lung, and trachea were used for RNA-Seq to obtain a comprehensive annotation of protein-coding genes. Animal care and experimental protocols were approved by the Northwestern Polytechnic University Ethics Committee Institutional Review Board (approval number 202301024).

The Illumina sequencing library was generated using the NEB Next® Ultra™ DNA Library Prep Kit (NEB, USA) and the sequencing was performed on the HiSeq 2000 platform. Raw sequencing reads were filtered for adapter sequences, low-quality reads, and trimmed using Fastp v0.20^9^ with default parameters and yielding a total of 189.18 Gb of clean short reads. To generate HiFi reads, we followed PacBio’s standard protocol (Pacific Biosciences, CA, USA), which yielded 74.23 Gb cleaned long reads with the PacBio Sequel II platform. For Hi-C library construction, we followed the standard protocol described in a published study^10^. The HiSeq X Ten platform was utilized for sequencing, producing a total of 234 Gb of cleaned reads with Fastp v0.20^9^.

To obtain RNA sequences from multiple tissues, the TRIzol kit (TIANGEN, Cat # DP424, China) was used to extract RNA. After constructing libraries for each sample, the Illumina NovaSeq 6000 platform was used for sequencing, Fastp v0.20^9^ was used to clean the raw data and each sample obtains more than 6 G of data.

### Chromosome level genome assembly of pig-nosed turtle

**Fig. 1:**
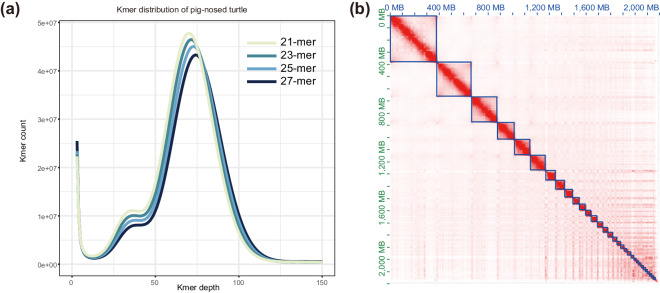
Genome assembly of pig-nosed turtle. (**a**) Different *k*-mer (k = 21, 23, 25, 27) distribution of the pig-nosed turtle genome. Genome size inferred by k num divided by k depth. The range of estimated genome size from 2.15 to 2.17 Gb for pig-nosed turtle. (**b**) Hi-C linkage density heat map of the pig-nosed turtle. The x-axis and y-axis represent genomic positions. Red dots indicate regions with a high density of paired reads, suggesting that they are more likely to be on the same chromosome.

To estimate the genome size, we performed *k*-mer analysis based on Illumina sequencing reads. The optimal *k*-mer value was calculated by Jellyfish v2.2.10^11^ and GenomeScope v2.0^12^ was used to estimate the genome size for corresponding different *k* values. We utilized four distinct *k*-mer values—21, 23, 25, and 27 and the corresponding genome size estimates are 2.16, 2.15, 2.17, and 2.16 Gb, respectively. (Fig. 1a). The HiFi reads were assembled into contigs using Hifiasm v0.16.1^13^ with default parameters. The resulting contig-level assembly spanning 2.18 Gb comprised 155 contigs with an N50 of 126.6 Mb (Table 1). The size of the genome assembly is very close to the results predicted by *k*-mer analysis. Then, Hi-C sequencing reads were aligned to the contig assembly utilizing BWA v0.7.12^14^. Following this, we used YaHS v1.1a-r3^15^ to generate the scaffolded genome assembly. JuiceBox v.1.11.08^16^ was used to visually correct the assembly based on the strength of chromosomal interactions. Contigs with no obvious interaction relationship were processed as individual scaffolds. Contigs assigned to one chromosome were joined with 200 ‘N’ to build the final chromosome-level genome assembly. Reflecting the 2N = 68 karyotype reported in study^17^, our final assembly comprised 34 chromosomes with an anchoring rate of 99.6% (Fig. 1b).

**Table 1:** Statistics of the genome assembly.

Term	Contig assembly		Hi-C assembly	
	Size (bp)	Number	Size (bp)	Number
N90	20,314,305	24	26,117,499	21
N80	40,037,570	17	40,688,678	15
N70	50,809,117	12	54,081,386	10
N60	70,227,638	8	76,607,989	7
N50	126,464,372	5	130,115,908	5
Max length (bp)	383,128,740	—	383,128,740	—
Total size (bp)	2,180,686,862	—	2,180,689,262	—
Total number	—	152	—	143

### Assessment of the genome assembly

**Fig. 2:**
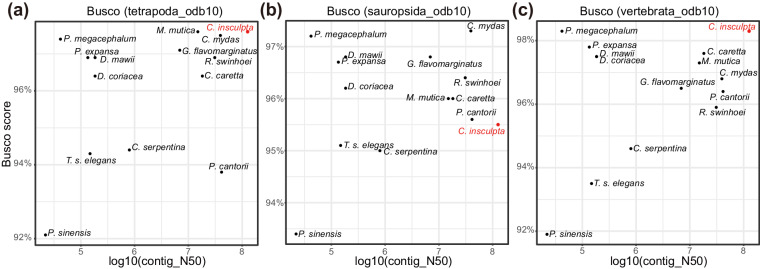
Quality statistics of genomes of the representative turtles. **(a**–**c**) The x-axis indicates the contig N50 of the genome assembly, while the y-axis indicates the genome BUSCO scores based on the tetrapoda_odb10, sauropsida_odb10 and vertebrata_odb10 lineage databases, respectively.

**Fig. 3:**
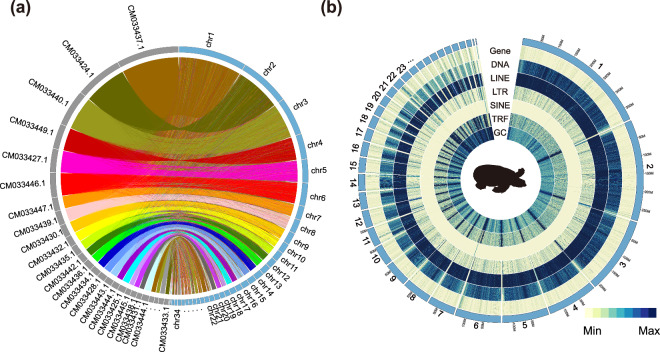
Overview of the pig-nosed turtle genome. (**a**) Synteny alignment in pig-nosed turtles and *R. swinhoei*. The blue circle represents chromosomes of pig-nosed turtle, while the grey circle represents chromosomes of *R. swinhoei*. (**b**) The densities of protein coding genes and different types of repeat sequences density and GC content are shown in the inner rims with a window size of 10 Mb. The numbers 1 to 34 correspond to the chromosomes of the pig-nosed turtle.

The assembled genome was evaluated for completeness using BUSCO v5.5.0^18^. The analysis revealed a genome completeness score of 97.6% including 97.2% single-copy orthologous genes using the tetrapoda_odb10 lineage database. The BUSCO scores based on the sauropsida _odb10 (95.5%) and vertebrata_odb10 (98.3%) lineage databases were also all above 95%. We compared the genome completeness by Busco score and contig N50 length with 13 published turtle genomes, Including *Pelodiscus sinensis* (GCF_000230535)^19^, *Chelonia mydas* (GCF_015237465)^19^, *Rafetus* *swinhoei* (GCA_019425775.1)^20^, *Caretta caretta* (GCF_023653815)^21^, *Pelochelys cantorii* (GCA_032595735.1)^22^,*Dermochelys coriacea* (GCF_009764565.3)^23^, *Chelydra serpentina* (GCA_018859375.1)^24^, *Dermatemys mawii* (GCA_007922305.1), *Gopherus flavomarginatus* (GCF_025201925.1), *Mauremys mutica* (GCF_020497125)^25^, *Platysternon megacephalum* (GCA_003942145.1)^26^, *Trachemys scripta elegans* (GCF_013100865.1)^27^ and *Podocnemis expansa* (GCA_007922195.1)^28^ from NCBI. Our assembly is comparable to the achievements observed in other genomes (Fig. 2, Table 2). Furthermore, to assessed the continuity of the assembly, we performed a genome alignment between the pig-nosed turtle and the closely related Yangtze giant softshell turtle using LAST v1282^29^ with parameters “-P 20 -i 2 G -m 10” to identify syntenic regions. The genome alignment revealed intact synteny between the two genomes, further validating the quality of the pig-nosed turtle genome assembly (Fig. 3a).

**Table 2:** Statistics of the genome assembly on the presence of conserved BUSCO orthologs.

Library	tetrapoda_odb10	sauropsida_obd10	vertebrata_obd10
Complete BUSCOs (C)	5,180	7,139	3,297
Complete and single-copy BUSCOs (S)	5,159	7,075	3,270
Complete and duplicated BUSCOs (D)	21	64	27
Fragmented BUSCOs (F)	23	52	18
Missing BUSCOs (M)	107	289	39
Total BUSCO groups searched	5,310	7,480	3,354
Summarize	97.60%	95.5%	98.3%

### Genome annotation

A comprehensive genome annotation was performed for pig-nosed turtle, focusing on repetitive sequences, protein-coding genes, and functional predictions. For repetitive sequence annotation, approximately 1 Gb of repetitive regions in the assembled sequence were detected by *de novo* annotation and homology annotation. For *de novo* annotation, Tandem Repeats Finder (TRF) v4.0.9^30^ was employed to annotated simple tandem repeats. The parameters used were “2 5 7 80 10 50 2000 -d -h -ngs”. Additionally, we utilized RepeatModeler v1.0.11 (http://www.repeatmasker.org/RepeatModeler/) to *de novo* annotate transposable elements. To perform homology-based annotation of transposable elements, we employed with RepeatMasker v4.0.6^31^ and RepeatProteinMask v1.0.8^31^ with parameters “-engine ncbi -noLowSimple -pvalue 1e-04”. The annotated repetitive sequence were then converted to lowercase letters in the genome using BEDtools v2.29.2^32^ to generate soft-masked sequences. The repetitive sequences account for 46.86% of the total genome length and include 310 Mb of DNA elements (14.23% of total length), 346.89 Mb of long interspersed nuclear elements (LINEs) (15.91% of total length), 25.38 Mb of short interspersed nuclear elements (SINEs) (1.16% of total length), and 121.3 Mb of long terminal repeats (LTRs) (5.56% of total length) (Fig. 3b and Table S1).

For protein-coding gene prediction, we employed a combination of three methods: *de novo* prediction, homology-based prediction, and transcript-based prediction. For *de novo* prediction, we used Augustus v2.5.5^33^. For homology-based prediction, we downloaded protein sets of closely related species mentioned above. Gene structures were predicted based on these homology proteins applied by miniport v0.12-r237^34^. Additionally, we obtained protein sets of the pig-nosed turtle using TransDecoder v5.5.0 based on RNA-Seq. The detailed steps include that SPAdes v3.1.1^35^ was performed transcriptome assembly and then TransDecoder v5.5.0 (https://github.com/TransDecoder/) used to predict protein structure. We aligned it to pig-nosed turtle genome using BLAST v2.6.0^36^. Gene structures were predicted using GeneWise v2.2.0^37^. The results of the three prediction methods were integrated into a final gene set using EVidenceModeler v1.1.1^38^. A total of 19,175 protein-coding genes were annotated in the pig-nosed turtle genome assembly, with a BUSCO completeness of 97.6% using tetrapoda_odb10 lineage database.

Functional annotation of proteins was applied by comparing protein sequences with public databases including Gene Ontology (GO) annotations (http://geneontology.org/), Cluster of Protein Orthologous Groups (COG: https://www.ncbi.nlm.nih.gov/COG/), Swiss-Prot (www.uniprot.org), TrEMBL (www.uniprot.org) and non-redundant proteins (NR: https://ftp.ncbi.nlm.nih.gov/blast/db) and Kyoto Encyclopedia of Genes and Genomes (KEGG: https://www.kegg.jp/) and InterPro. 99.70% proteins and were annotated in these databases (Table 3).

**Table 3:** Statistics of the functional annotation of protein-coding genes.

Term	Number	Percent (%)
InterPro	17,917	93.44
GO	12,412	64.73
KEGG	15,036	78.41
Swissprot	18,589	96.94
TrEMBL	18,954	98.85
COG	6,977	36.39
NR	19,074	99.47
Annotated genes	19,117	99.70
Missing genes	58	0.30
Total genes	19,175	—

## Data Records

All sequencing data and genome assembly have been deposited in the National Center for Biotechnology Information (NCBI) database (https://www.ncbi.nlm.nih.gov/bioproject/PRJNA1037723), which include SRR26796054^39^, SRR26796055^40^, SRR26796056^41^, SRR26796057^42^, SRR26796058^43^, SRR26796059^44^, SRR267960^45^, SRR26796061^46^ in SRA and JAWWUY000000000^47^ in GenBank. The genome annotation deposited in the Figshare database (10.6084/m9.figshare.24630915)^48^.

## Technical Validation

The integrity of the extracted DNA was checked by agarose gel electrophoresis, and the concentration of DNA was determined using Qubit fluorometer using the 1 × dsDNA HS kit. Contig N50 (126.6 Mb) and BUSCO score (97.60%) are much higher quality than genomes of other turtles (Fig. 2). Higer anchoring rate (99.6%) and a strong linear relationship between the genome assembly of pig-nosed turtle and Yangtze giant softshell turtle validating that we obtained a contiguous pig-nosed turtle genome.

### Supplementary information

supplementary information

## Data Availability

The data analyses were performed all software and parameters were mentioned in Methods. The core code is available at https://github.com/YuXuanLiua/pig-nosed-turtle/.
