# Supplementary material for: A chromosome-level genome assembly of the pig-nosed turtle (Carettochelys insculpta)
Source: Sci Data. 2024 Mar 23;11:311. doi: 10.1038/s41597-024-03157-8 (PMC10960847; doi:10.1038/s41597-024-03157-8)
Supplement: Supplementary file 1 — supplementary information [file 41597_2024_3157_MOESM1_ESM.docx]

**Supplementary Information for**

**A chromosome-level genome assembly of the pig-nosed turtle (*Carettochelys insculpta*)**

**Ye Li**^1,*^, **Yuxuan Liu**^1,*^, **Jiangmin Zheng**^1,*^, **Baosheng Wu**^1,2,^**^*^**, **Xinxin Cui**^1,*^, **Wenjie** **Xu**^1^, **Chenglong Zhu**^1^, **Qiang Qiu**^1,#^, **Kun Wang**^1,#^

^1^ Shaanxi Key Laboratory of Qinling Ecological Intelligent Monitoring and Protection, School of Ecology and Environment, Northwestern Polytechnical University, Xi’an 710072, China.

^2^ Guangdong Key Laboratory of Animal Conservation and Resource Utilization, Institute of Zoology, Guangdong Academy of Sciences, Guangzhou, 510260, China.

^*^ These authors contributed equally to this work.

^#^ Corresponding author. Email: wk8910@gmail.com (K. W.); qiuqiang@lzu.edu.cn (Q. Q.).

**This file includes:** **Supplementary Table 1.**

**Supplementary Table 1. Summary of repetitive sequences in pig-nosed turtle.**

|  | Repbase TEs | | TE proteins | | De novo | | Combined TEs | |
| --- | --- | --- | --- | --- | --- | --- | --- | --- |
| Type | Length  (bp) | Percent in  genome  (%) | Length  (bp) | Percent in  genome  (%) | Length  (bp) | Percent in  genome  (%) | Length  (bp) | Percent in  genome  (%) |
| DNA | 102,270,227 | 4.69 | 161,591 | 0.01 | 283,605,093 | 13.01 | 310,207,527 | 14.23 |
| LINE | 293,188,673 | 13.44 | 204,919,638 | 9.4 | 292,732,538 | 13.42 | 346,895,496 | 15.91 |
| SINE | 16,460,199 | 0.75 | 0 | 0 | 14,772,443 | 0.68 | 25,381,747 | 1.16 |
| LTR | 81,929,718 | 3.76 | 13,822,863 | 0.63 | 101,948,003 | 4.68 | 121,300,058 | 5.56 |
| Other | 7,001,938 | 0.32 | 5,334 | 0 | 18,356,333 | 0.84 | 19,716,622 | 0.90 |
| Unknown | 6,355,441 | 0.29 | 0 | 0 | 236,385,470 | 10.84 | 241,039,968 | 11.05 |
| Total | 508,659,977 | 23.33 | 218,875,702 | 10.04 | 930,185,802 | 42.66 | 1,021,763,536 | 46.86 |
